# Immediate effect of physical activity on the autonomic nervous system in individuals with autism spectrum disorders of different age groups: a randomised trial

**DOI:** 10.1136/bmjsem-2023-001822

**Published:** 2024-04-11

**Authors:** Mohamed M Ahmed, Motaz Alawna, Ahmed S A Youssef, Wafaa Mahmoud Amin, Ramzi Abdu Alajam, Walaa E Morsy, Esraa Fayed, Ayman A Mohamed

**Affiliations:** 1 Department of Physical Therapy, College of Applied Medical Sciences, Jazan University, Jazan, Jazan, Saudi Arabia; 2 Department of Basic Science for Physical Therapy, Faculty of Physical Therapy, Beni-Suef University, Beni Suef, Egypt; 3 Department of Health Sciences, Faculty of Graduate Studies, Arab American University, Jenin, Palestine, State of; 4 Department of Basic Science for Physical Therapy, Faculty of Physical Therapy, Cairo University, Giza, Egypt; 5 Department of Pediatrics, Faculty of Physical Therapy, Cairo University, Giza, Egypt; 6 Department of Basic Science for Physical Therapy, Faculty of Physical Therapy, Nahda University, Beni Suef, Egypt; 7 Department of Basic Science for Physical Therapy, Faculty of Physical Therapy, Galala University, Suez, Egypt

**Keywords:** Exercise physiology, Aerobic fitness, Fit

## Abstract

**Background:**

Autism spectrum disorder (ASD) is one of the most complex neurodevelopmental disorders. It affects almost all human physiological systems. Individuals with ASD often display dysregulation in their autonomic nervous system (ANS), which may elicit differing effects across age groups. Also, studying the ANS missed several important parameters related to ANS. Studying the ANS is crucial in developing adaptive behavioural strategies and maintaining communication abilities and social behaviours. Thus, this study compared the immediate effect of physical activity on the ANS in individuals with ASD in different age groups.

**Methods:**

200 participants (106 males and 94 females) took part in a double-blinded randomised design. All participants were divided into four groups according to their age (4–7, 7–10, 10–13 and 14–18 years old). Participants performed a 60 min treadmill walk. The main outcome measurements were heart rate (HR), saturation of peripheral oxygen (SpO_2_), respiratory rate (RR) and end-tidal carbon dioxide (etCO_2_).

**Results:**

Before the study, there were non-significant differences between groups in their physical characteristics (body mass index, Childhood Autism Rating Scale, physical activity level, both parents’ existence, aerobic capacity and gender) (p>0.05). At baseline measurements, there were non-significant differences between all groups for all outcome measurements (p>0.05). Immediately after physical activity, there was significant difference between group 1 and other groups (p<0.05), while all other differences were non-significant (p>0.05). At the follow-up (after 15 min of rest), group 1 maintained significant differences with the other groups for all outcome measurements (p<0.05), while there were non-significant differences between the other three groups (p>0.05).

**Conclusion:**

This study revealed that the SpO_2_ significantly decreased immediately after the physical activity, while HR, RR and etCO_2_ significantly increased immediately after physical activity in comparison to the baseline measurements. Contrary to other ANS parameters (SpO_2_, RR and etCO_2_), HR in early ages (4–7 years old) was higher after physical activity and remained elevated longer than other ages. The early ages (4–7 years old) take more time to return to the normal status of ANS parameters including SpO_2_, HR, RR and etCO_2_.

**Trial registration number:**

NCT05725733.

**WHAT IS ALREADY KNOWN ON THIS TOPIC**

- The autonomic nervous system may demonstrate a varying response to physical activity in individuals with autism spectrum disorders (ASD) between 4 and 18 years old.
- Age may affect the response of individuals with ASD to physical activity or not.

**WHAT THIS STUDY ADDS**

- Individuals with ASD from 4 to 18 years old have similar responses in saturation of peripheral oxygen, respiratory rate and end-tidal carbon dioxide parameters.
- Heart rate of individuals with ASD in early ages (4–7 years old) shows higher response after physical activity and remains elevated longer than other ages.

**HOW THIS STUDY MIGHT AFFECT RESEARCH, PRACTICE OR POLICY**

- These findings recommend that the physical activity programmes should be individually designed for individuals with ASD of different ages.

## Introduction

Autism spectrum disorder (ASD) is considered one of the most complex neurodevelopmental disorders, affecting several bodily systems and organs.^1^ Several epidemiological studies in different countries (Denmark,^1^ Finland^2^ and the UK^3^) demonstrated that the incidence of ASD has increased since 2000 until now. The WHO has suggested that ASD occurs in around 1 in every 100 children^4^; however, the Centers for Disease Control and Prevention’s Autism and Developmental Disabilities Monitoring Network reports that ASD may occur in as many as 1 in every 36 children.^5^ They found that ASD occurs in both genders with a higher incidence in males than females, nearly four times more prevalent among boys than among girls, with ratios 4:1–8:1.^3 6 7^ In South Korean children aged 7–12 years, the incidence of ASD was predicted to be 2.64%, with 1.89% in the overall population sample.^6^ Another study conducted to estimate the incidence of ASD in children aged 9–10 years in South Thames, UK, found that ASD occurs at a rate 38.9 per 10 000.^3^

Studying the autonomic nervous system (ANS) is crucial in developing adaptive behavioural strategies, and maintaining communication abilities and social behaviours.^8^ First, the newborn uses simple brainstem-visceral paths via ingestive behaviours as the fundamental mechanism to adjust the physiological state. Then, as cortical regulation of the brainstem advances throughout the initial years of life, reciprocal social behaviour relocates feeding as the chief regulator of physiological state.^9^ The ANS can decrease vocalisations and facial expressions, and increase the activities of the parasympathetic nervous system which are essential for any behavioural approach.^8^ Furthermore, the activation of the sympathetic nervous system (‘flight or fight’ response) during social encounters may lead to ‘threat-like’ responses.^8^ Ellenbroek and Sengul^10^ stated that respiratory and cardiovascular responses are different in individuals with ASD with more increase in their baseline data.

The response of the heart varies with ageing in healthy people.^11^ Individuals with ASD have varying responses of the heart to external stimuli, too. Individuals with ASD have higher heart rate (HR) at rest^12–15^ and sleep^16^ than typical children (TD) children due to their higher fear of unknown examination procedures or having higher rate of anxiety than TD. Individuals with ASD have a declined HR response to social activity, like social role-playing,^17^ community speaking,^14^ unfamiliar partner,^15^ and public speech,^12^ or physical activity^18^; they attributed this decline to the lowered degree of attention that children with ASD have in comparison to TD children. In contrast, other studies denied these hypotheses and reported that there is non-significant difference in HR between children with ASD in comparison to TD children during the performance of the Stroop Color and Word task^14^ and social or non-social images.^19^

The current studies that investigated the effect of ANS to physical activity among individuals with ASD have been mainly conducted in adolescents with ASD. These studies focused mainly on the maladaptive behaviours among adolescents with ASD. These studies did not investigate the effect of physical activity on ANS response. The current studies did not investigate the effects of physical activity on HR adaptation and aerobic capacity in individuals with ASD, or difference in the response of individuals with ASD of different ages to physical activity. One study investigated the effect of children with ASD on physical activity.^18^ They found that children with ASD have low cardiorespiratory response to physical activity. Despite the valuable results of this study, this study recruited only male children aged 10 years and did not compare this response in individuals with ASD of different ages.

To the best of our knowledge, few studies have measured the change in respiratory parameters among children with ASD and yet no study investigated the complete respiratory profile of individuals with ASD. The complete respiratory profile of any individual should contain respiratory rate (RR) and rhythm in addition to changes in gas pressure and volumes (oxygen and carbon dioxide), and no study has brought these aspects surrounding respiratory profiles together. Some studies found that children with ASD had respiratory dysrhythmias like Biot’s respiration and Cheyne-Stokes respiration, higher respiratory sinus arrhythmia reactivity.^20–22^ Others found that aerobic capacity, effort duration and maximal speed were significantly lower than in TD children. The data related to carbon dioxide have not been demonstrated in the literature yet despite its important role in recognising respiratory problems. Therefore, a measurement of the complete respiratory profile is still needed.

The ANS activity among children with ASD is still not fully understood. The response of the ANS to physical activity was barely studied, despite the major role of the ANS in improving the physical abilities of children with ASD. Also, despite the variability that exists between children, adolescents and adults with ASD, most studies did not investigate the variation between these age groups. Thus, this study was conducted to compare the immediate effect of physical activity on the ANS in individuals with ASD of different age groups. This study measured saturation of peripheral oxygen (SpO_2_), HR, RR and end-tidal carbon dioxide (etCO_2_) parameters in different age groups among individuals with ASD.

## Materials and methods

### Study design

This study followed a double-blinded randomised trial design. Data were collected from public hospitals and private clinics in Jazan, Saudi Arabia. All measurements were conducted at the physical therapy laboratories of Jazan University, Saudi Arabia. Each parent signed the consent form after giving detailed information about the study and procedures. The data were collected from April 2023 to July 2023. This study was registered at https://www.clinicaltrials.gov/ website (registration number: NCT05725733).

#### Power analysis

The sample size was predetermined by performing a prior power test via the G*Power program. The data entered were repeated measures analysis of variance (ANOVA): fixed effects, main effects and interactions; a medium effect size of 0.25; a power of 80%, a number of df of 4; 4 groups; and 1 covariant. According to the power analysis, the suitable sample size was 196 participants. We added four participants to compensate for any dropouts. We used a minimum power of 80% or more as it is accepted in most of the previous studies.^23^

#### Participant recruitment procedures

Initially, we recruited children with ASD aged 4–18 years to this study. Then, they were scanned for the inclusion and exclusion criteria. The inclusion criteria included (a) having a specialised medical diagnosis of ASD done by each case following physician, (b) having no recent history of participation in physical activity or physical activity programmes, (c) having an initial medical check-up to determine fitness and safety to participate in study’s physical activity programme and (d) having a mild severity (scores between 30 and 36.5) ASD assessed using the Childhood Autism Rating Scale (CARS).^24 25^ The exclusion criteria included (a) having any intellectual disability (IQ≤70) assessed by the Wechsler Intelligence Scale for Children-Fourth Edition^26^ according to the Diagnostic and Statistical Manual of Mental Disorders-Fourth Edition and International Classification of Diseases 10th Revision criteria, (b) having any comorbid medical or psychiatric disorders, pervasive developmental disorders or attention-deficit hyperactivity disorders, (c) taking any medications that affect the cardiovascular system, respiratory system, arousal and balance like antidepressants, beta-adrenergic drugs, antiepilepsy drugs, etc and (d) having any contraindications to physical activity like fracture, dislocations, severe seizures, etc.^24 25^

#### Randomisation

We randomly chose participants from the screened individuals in each group using the Excel software. The participants were distributed to four groups (4–7 years old (preschool), 7–10 years old (child), 10–13 years old (preteen or teen) and 13–18 years old (teenagers)). The target number of participants was 50 participants in each group. Once this number limit was reached, we stopped the recruiting process for this group. Randomisation and patient allocation were performed before baseline measurements. This study used a sealed opaque envelope to mask the allocation code/sequence to all participants. The randomisation procedure was directed by a college staff member who was blind to the trial’s aim and procedures and was not involved in this study. After the randomisation and allocation processes, two handheld participants were included in this study.

### Assessment procedures

#### Physical characteristic measurements

The measurements of physical characteristics were performed according to the following order: (a) Anthropometric measurements (height and weight) were performed at the beginning. The body mass index (BMI) was calculated as weight divided by height squared (kg/m^2^). (b) The activity levels of children with ASD and controls in daily life were assessed using the Vineland Adaptive Behavior Scale, which is a standardised evaluation method that uses semidesigned interview to evaluate adaptive behaviour and support the diagnosis of ASD, intellectual and developmental disabilities and developmental delays.^27^ (c) The severity of symptoms was evaluated using the CARS. The CARS is a valid and reliable rating scale to assess the severity of symptoms in individuals with ASD.^28^ The CARS scale was applied by an experienced psychologist who was blinded to study procedures. (d) The aerobic capacity was assessed using VO_2max_. The VO_2max_ is the gold standard for measuring cardiorespiratory fitness.^29^ The VO_2max_ of each participant was measured by easy walking 3 km/hour on a treadmill (Technogym USA, New York, USA). Then, the velocity and the incline of the treadmill were raised by 0.8 km/hour—1% or 2% each minute until tiredness. Throughout this maximal test, gas exchanges were assessed constantly by an ergospirometer (MedicalExpo, Marseille, France).

#### ANS measurements

ANS measurements were performed under the same conditions for all groups. The measurements were performed in a quiet room with a temperature of 23–25°C. All measurements were performed in the presence and under the parent’s observation of the therapist and parents. The parents could access the child at any time during study procedures. During all measurements, the children were seated on a reclined chair with a head support to decrease neck muscle tension. The study procedures were performed between 10:30 and 15:30. Before starting the procedure, participants were allowed to rest for 15 min after coming to the laboratories.

All participants underwent a preparation session to get familiar with physical activity and measurement tools. The main outcome measurements were SpO_2_, HR, RR and etCO_2_. We studied the etCO_2_ because it allows continuous and non-invasive estimation of cardiac activity.^30^ The measurements were performed before the physical activity session (pretest), after the physical activity session (1 min post-test) and 15 min after the physical activity session (follow-up). Each measurement was taken three times, and the average was taken for analysis as minute averages for each study participant.

##### Respiratory parameter measurements

The SpO_2_ was used to assess the amount of oxygen-carrying haemoglobin in blood to the periphery which is important for functional activities such as walking and climbing. A Capnostream 35 Portable Respiratory Monitor (Medtronic, Minnesota, USA) was used to measure the etCO_2_, SpO_2_ and RR. This device uses respiratory inductive plethysmography (RIP) technology to measure respiratory activity. It gives a real-time measurement of individual status by assessing etCO_2_, SpO_2_ and RR. The American Academy of Sleep Medicine has endorsed the use of this technology for assessing respiratory activity.^31^ The RIP technology standards, values and applications have been established by J Scott Cardozo.^31^ The RIP sensor consists of an inductive coil made of shielded interlaced wires or seamed in a sine wave or zigzag pattern on stretched two belts. The first belt was positioned on the rib cage and the second belt was positioned on the abdomen. Both belts were adjusted to the chest size.

##### HR measurement

This study used HR to focus on the average beats per minute as an indicator of heart response to physical activity. An unobtrusive wearable device was used to collect cardiac ECG data. This platform consists of three units: the biosignal sensor unit (BSU), the video mobile unit (VMU) and the central unit (CU). These three units work together to efficiently gather cardiac activity information. The BSU consists of electroencephalogram (EEG) and electrocardiography (ECG) wearable hardware. The VMU consists of two wired cameras for scene recording to allow clinicians to observe the child’s behaviour and actions during treatment or physical activities. The CU consists of a workstation that allows online and offline physiological signal management. Also, the CU allows clinicians to synchronise, store and retrieve the acquired data. The ECG wearable device technology belongs to the biosignal unit and consists of an IFC-CNR wireless ECG chest belt. This wearable device was validated by Solar *et al*^32^ on healthy controls by comparing it to the gold standard Holter device (ELA Medical, Milan, Italy).^32^ The device is built on the CE-certified Shimmer^33^ wireless base module. This module is small (50×25×23 mm) and lightweight (30 g). The device also includes transducing, amplification and single preprocessing blocks for signal conditioning. The device has a 2 GB SD card for data transmission modules and data storage. The output signal is sampled at 200 Hz and has an A/D resolution of 12 bits. A Chipcon CC2420 radio transceiver and GigaAnt 2.4 GHz Rufa antenna (central station transmission ranges up to 30 m) was used for sensor wireless communication.

#### Physical activity programme

The physical activity programme lasted for 60 min. The participant wore comfortable clothes and sports shoes. The participant was watched carefully during physical activity and if any discomfort or fatigue signs were noticed, physical activity was stopped immediately. The physical activity area was prepared to be safe and suitable for participants.

Each participant performed a treadmill walk. The participant was positioned on the treadmill with the help of parents and held the sidebars. A Velcro strap was used to ensure safety during physical activity. The strap was attached to the waist of the participant. If the participant stopped walking or could not follow the treadmill speed, the Velcro strap released, and the treadmill stopped automatically. During warming up, the velocity was increased gradually up to 3 km/hour because this velocity was reported as an average walking velocity for individuals under 30 years old^34^ and lasted for 5 min. After this, the velocity was increased gradually up to 5–6 km/min under the supervision of the parents and the researcher as much as possible. The participant ran 10 min at this speed. The cooling down period was at the same velocity and time as the warm-up period and gradually stopped. After finishing physical activity, the participant moved to sit on a chair or lie on a bed to start the measurement procedures.

### Statistical analysis

Statistical analysis was performed in the form of parametric tests which included repeated measures ANOVA. Data were collected at the baseline, after physical activity and 15 min after physical activity (follow-up). The statistical analysis compared results within each group and between the four groups. Power analysis using an ANOVA (repeated measures, within-between groups), eight groups were conducted in G*Power to determine sufficient sample size using an alpha of 0.05, a power of 0.90 and a medium effect size (f=0.25).^35^ Based on the aforementioned assumptions, the desired sample size is 25 in each group. A power of 80% and more is acceptable in most studies.^36–38^ The physical characteristics of participants were compared using a t-test for BMI, VO_2max_ and CARS, and a Wilcoxon test for physical activity level, and parents’ existence. The normal distribution of data across groups was confirmed by using the Shapiro-Wilk test. The SPSS V.27 (SPSS) program was used to conduct statistical analysis in this study. All data were collected from a pairwise comparison table.

## Results

The physical characteristics of the participants included BMI, CARS, VO_2max_, physical activity level, both parents’ existence and gender. The main outcome measures included SpO_2_ (%), HR (pulse per minute (ppm)), RR (breaths per minute (bpm)) and etCO_2_ (%). All outcome measures were performed at baseline, immediately after physical activity (1 min after test) and at follow-up (at follow-up (after 15 min of rest)).

### The physical characteristics of the participants

At baseline, there were non-significant differences between all groups in the BMI, CARS, VO_2max_, physical activity level, both parents’ existence and gender (p>0.05). The average BMI in groups 1, 2, 3 and 4 was 21.12±1.03, 22.01±1.21, 21.98±1.32 and 22.03±1.64, respectively. The average CARS in groups 1, 2, 3 and 4 was 31.32±2.12, 32.02±1.31, 32.24±3.31 and 32.92±2.31, respectively. The average VO_2max_ in groups 1, 2, 3 and 4 was 52±2.34, 51±1.58, 52±3.15, and 53±2.87, respectively. The number of males in groups 1, 2, 3 and 4 was 26, 27, 26 and 27, respectively, while the number of females in groups 1, 2, 3 and 4 was 24, 23, 24 and 23, respectively. The physical characteristics of participants and the descriptive statistics are shown in summary tables 1 and 2, respectively.

**Table 1:** Physical characteristics of the participants in this study

		Group 1	Group 2	Group 3	Group 4	P value
BMI		21.12±1.03	22.01±1.21	21.98±1.32	22.03±1.64	>0.05
CARS		31.32±2.12	32.02±1.31	32.24±3.31	32.92±2.31	>0.05
Physical activity level		Sedentary	Sedentary	Sedentary	Sedentary	>0.05
Both parents’ existence		Both	Both	Both	Both	>0.05
Aerobic capacity (VO_2max_)		52±2.34	51±1.58	52±3.15	53±2.87	>0.05
Gender	Male	26	27	26	27	>0.05
	Female	24	23	24	23	

BMI, body mass index; CARS, Childhood Autism Rating Scale.

**Table 2:** Descriptive statistics of participants in all groups

Age group, years	Mean	SD	n
SpO_2_ pre			
G1, 4–7	94.84	1.01	50
G2, 7–10	94.22	2.80	50
G3, 10–13	94.68	1.11	50
G4, 13–18	93.90	4.72	50
Total	94.41	2.85	200
SpO_2_ immediately after			
G1, 4–7	90.54	0.81	50
G2, 7–10	90.24	0.82	50
G3, 10–13	90.08	0.75	50
G4, 13–18	90.14	0.80	50
Total	90.25	0.81	200
SpO_2_ 15 min post			
G1, 4–7	92.12	0.74	50
G2, 7–10	91.70	0.81	50
G3, 10–13	91.46	0.73	50
G4, 13–18	91.70	0.73	50
Total	91.74	0.78	200
Heart rate pre			
G1, 4–7	95.46	12.25	50
G2, 7–10	94.30	14.79	50
G3, 10–13	96.86	13.08	50
G4, 13–18	93.88	9.915	50
Total	95.12	12.59	200
Heart rate immediately after			
G1, 4–7	117.36	13.93	50
G2, 7–10	113.26	15.90	50
G3, 10–13	114.88	13.73	50
G4, 13–18	115.04	13.23	50
Total	115.13	14.20	200
Heart rate 15 min post			
G1, 4–7	107.22	12.67	50
G2, 7–10	96.02	14.71	50
G3, 10–13	99.60	13.70	50
G4, 13–18	99.72	12.57	50
Total	100.64	13.95	200
Respiratory rate pre			
G1, 4–7	15.42	1.23	50
G2, 7–10	15.42	1.23	50
G3, 10–13	15.42	1.23	50
G4, 13–18	15.42	1.23	50
Total	15.42	1.22	200
Respiratory rate immediately after			
G1, 4–7	24.50	2.89	50
G2, 7–10	23.16	2.70	50
G3, 10–13	22.04	2.94	50
G4, 13–18	21.84	3.21	50
Total	22.88	3.11	200
Respiratory rate 15 min post			
G1, 4–7	19.14	2.06	50
G2, 7–10	17.92	1.88	50
G3, 10–13	17.02	2.30	50
G4, 13–18	16.36	2.89	50
Total	17.61	2.52	200
etCO_2_ pre			
G1, 4–7	38.90	4.00	50
G2, 7–10	38.90	4.00	50
G3, 10–13	39.44	4.23	50
G4, 13–18	40.24	4.49	50
Total	39.37	4.19	200
etCO_2_ immediately after			
G1, 4–7	51.64	3.63	50
G2, 7–10	51.60	3.93	50
G3, 10–13	53.18	5.44	50
G4, 13–18	54.78	5.54	50
Total	52.80	4.86	200
etCO_2_ 15 min post			
G1, 4–7	44.56	3.90	50
G2, 7–10	40.88	3.99	50
G3, 10–13	41.56	4.22	50
G4, 13–18	42.22	4.45	50
Total	42.30	4.34	200

etCO_2_, end-tidal carbon dioxide; SpO_2_, saturation of peripheral oxygen.

### Between-group analysis

#### Baseline measurements

At baseline measurements, there were non-significant differences between all groups for all outcome measurements (p>0.05).

For SpO_2_, the mean difference between group 1 and group 2, group 1 and group 3, group 1 and group 4, group 2 and group 3, group 2 and group 4, and group 3 and group 4 was 0.54%, 0.24%, 0.84%, 0.30%, 0.30% and 0.60%, respectively.

For HR, the mean difference between group 1 and group 2, group 1 and group 3, group 1 and group 4, group 2 and group 3, group 2 and group 4, and group 3 and group 4 was 3.40 ppm, 1.44 ppm, 0.94 ppm, 1.96 ppm, 2.46 ppm and 0.50 ppm, respectively.

For RR, the mean difference between group 1 and group 2, group 1 and group 3, group 1 and group 4, group 2 and group 3, group 2 and group 4, and group 3 and group 4 was 0.22 bpm, 0.02 bpm, 0.02 bpm, 0.20 bpm, 0.20 bpm and 1.78 bpm, respectively.

For etCO_2_, the mean difference between group 1 and group 2, group 1 and group 3, group 1 and group 4, group 2 and group 3, group 2 and group 4, and group 3 and group 4 was 0.16%, 0.52%, 1.58%, 0.36%, 1.42% and 1.06%, respectively.

#### Immediately after physical activity (after 1 min)

Immediately after physical activity, there were non-significant differences between all groups for all outcome measurements (p>0.05) except for the HR. There was a significant difference between group 1 and other groups in HR (p<0.05).

For SpO_2_, the mean difference between group 1 and group 2, group 1 and group 3, group 1 and group 4, group 2 and group 3, group 2 and group 4, and group 3 and group 4 was 0.30%, 0.36%, 0.26%, 0.06%, 0.04% and 10%, respectively.

For HR, the mean difference between group 1 and group 2, group 1 and group 3, group 1 and group 4, group 2 and group 3, group 2 and group 4, and group 3 and group 4 was 8.68* ppm, 5.88 ppm, 4.28 ppm, 2.80 ppm, 4.40 ppm and 1.60 ppm, respectively.

For RR, the mean difference between group 1 and group 2, group 1 and group 3, group 1 and group 4, group 2 and group 3, group 2 and group 4, and group 3 and group 4 was 1.12 bpm, 0.48 bpm, 0.20 bpm, 0.64 bpm, 0.92 bpm and 28 bpm, respectively.

For etCO_2_, the mean difference between group 1 and group 2, group 1 and group 3, group 1 and group 4, group 2 and group 3, group 2 and group 4, and group 3 and group 4 was 0.04%, 1.54%, 2.00%, 1.58%, 2.04% and 0.46%, respectively.

#### At follow-up (after 15 min of rest)

At follow-up (after 15 min of rest), there were significant differences between group 1 and other groups for all outcome measurements (p<0.05) and there were non-significant differences between the other three groups (p>0.05).

For SpO_2_, the mean difference between group 1 and group 2, group 1 and group 3, group 1 and group 4, group 2 and group 3, group 2 and group 4, and group 3 and group 4 was 0.42*%, 0.66*%, 0.42*%, 0.24%, 0.05% and 0.12%, respectively.

For HR, the mean difference between group 1 and group 2, group 1 and group 3, group 1 and group 4, group 2 and group 3, group 2 and group 4, and group 3 and group 4 was 19.08* ppm, 12.94* ppm, 13.46* ppm, 6.14 ppm, 5.62 ppm and 0.52 ppm, respectively.

For RR, the mean difference between group 1 and group 2, group 1 and group 3, group 1 and group 4, group 2 and group 3, group 2 and group 4, and group 3 and group 4 was 1.64 bpm, 2.04 bpm, 2.70 bpm, 0.40 bpm, 1.06 bpm and 0.66 bpm, respectively.

For etCO_2_, the mean difference between group 1 and group 2, group 1 and group 3, group 1 and group 4, group 2 and group 3, group 2 and group 4, and group 3 and group 4 was 3.68*%, 3.00*%, 2.34*%, 0.68%, 1.34% and 0.66%, respectively.

**Figure 1:**
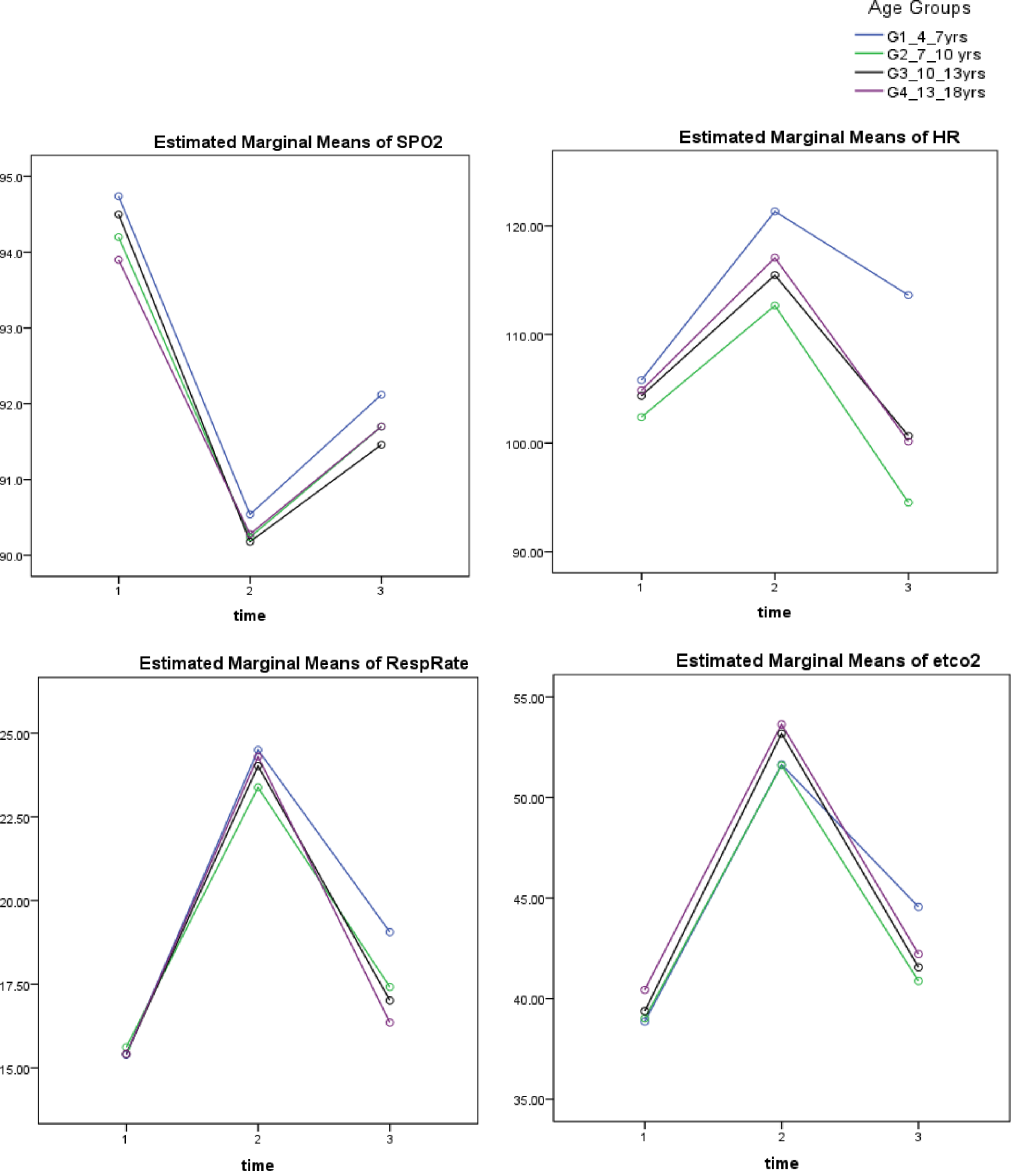
Between-group comparison. At baseline measurements, there were non-significant differences between all groups for all outcome measurements (p>0.05). Immediately after the physical activity, there were non-significant differences between all groups for all outcome measurements (p>0.05) except for the heart rate (HR). There was a significant difference between group 1 and other groups in HR (p<0.05). At follow-up (after 15 min of rest), there were significant differences between group 1 and other groups for all outcome measurements (p<0.05) and there were non-significant differences between the other three groups (p>0.05).

All between-group analyses at baseline, immediately after physical activity and at follow-up were shown in summary table 3 and online supplemental table 1, and figure 1.

10.1136/bmjsem-2023-001822.supp1 Supplementary data

**Table 3:** Between-group analysis for HR, SpO_2_, RR and etCO_2_

	Time	(I) Age group, years	(J) Age group, years	MD (I–J)	SE	P value	95% CI	
							Lower bound	Upper bound
Measure	Post-test	G1, 4–7	G2, 7–10	0.3	0.16	0.4	0.13	0.73
			G3, 10–13	0.36	0.16	0.17	0.07	0.79
			G4, 13–18	0.26	0.16	0.66	0.17	0.69
		G2, 7–10	G3, 10–13	0.06	0.16	1	0.37	0.49
			G4, 13–18	-0.04-	0.16	1	0.47	0.39
		G3, 10–13	G4, 13–18	-0.10-	0.16	1	0.53	0.33
	Follow-up	G1, 4–7	G2, 7–10	0.42*	0.15	0.04	0.02	0.82
			G3, 10–13	0.66*	0.15	0	0.26	1.06
			G4, 13–18	0.42*	0.15	0.04	0.02	0.82
		G2, 7–10	G3, 10–13	0.12	0.15	0.69	0.16	0.64
			G4, 13–18	0.05	0.15	1	0.40	0.4
		G3, 10–13	G4, 13–18	-0.24-	0.15	0.69	0.64	0.16
	Post-test	G1, 4–7	G2, 7–10	8.68*	2.61	0.01	1.73	15.63
			G3, 10–13	5.88	2.61	0.15	1.07	12.83
			G4, 13–18	4.28	2.61	0.61	2.67	11.23
		G2, 7–10	G3, 10–13	-2.80-	2.61	1	9.75	4.15
			G4, 13–18	-4.40-	2.61	0.56	-11.35-	2.55
		G3, 10–13	G4, 13–18	-1.60-	2.61	1	-8.55-	5.35
	Follow-up	G1, 4–7	G2, 7–10	19.08*	2.2	0	13.22	24.94
			G3, 10–13	12.94*	2.2	0	7.08	18.8
			G4, 13–18	13.46*	2.2	0	7.6	19.32
		G2, 7–10	G3,10–13	-6.14-*	2.2	0.03	12.00	-0.28-
			G4, 13–18	-5.62-	2.2	0.07	11.48	0.24
		G3, 10–13	G4, 13–18	0.52	2.2	1	5.34	6.38
	Post-test	G1, 4–7	G2, 7–10	1.12	0.58	0.34	0.43	2.67
			G3, 10–13	0.48	0.58	1	1.07	2.03
			G4, 13–18	0.2	0.58	1	1.35	1.75
		G2, 7–10	G3, 10–13	-0.64-	0.58	1	2.19	0.91
			G4, 13–18	-0.92-	0.58	0.7	2.47	0.63
		G3, 10–13	G4, 13–18	-0.28-	0.58	1	1.83	1.27
	Follow-up	G1, 4–7	G2, 7–10	1.64*	0.46	0	0.41	2.87
			G3, 10–13	2.04*	0.46	0	0.81	3.27
			G4, 13–18	2.70*	0.46	0	1.47	3.93
		G2, 7–10	G3, 10–13	0.4	0.46	1	0.83	1.63
			G4, 13–18	1.06	0.46	0.14	0.17	2.29
		G3, 10–13	G4, 13–18	0.66	0.46	0.92	0.57	1.89
	Post-test	G1, 4–7	G2, 7–10	0.04	0.93	1	2.44	2.52
			G3, 10–13	-1.54-	0.93	0.6	4.02	0.94
			G4, 13–18	-2.00-	0.93	0.2	4.48	0.48
		G2, 7–10	G3, 10–13	-1.58-	0.93	0.55	4.06	0.9
			G4, 13–18	-2.04-	0.93	0.18	4.52	0.44
		G3, 10–13	G4, 13–18	-0.46-	0.93	1	2.94	2.02
	Follow-up	G1, 4–7	G2, 7–10	3.68*	0.83	0	1.47	5.89
			G3, 10–13	3.00*	0.83	0	0.79	5.21
			G4, 13–18	2.34*	0.83	0.03	0.13	4.55
		G2, 7–10	G3, 10–13	-0.68-	0.83	1	2.89	1.53
			G4, 13–18	-1.34-	0.83	0.65	3.55	0.87
		G3, 10–13	G4, 13–18	-0.66-	0.83	1	-2.87-	1.55

CI denotes confidence interval for difference. P value denotes significance level.

*The mean difference is significant at the 0.05 level.

etCO_2_, end-tidal carbon dioxide; HR, heart rate; MD, mean difference; RR, respiratory rate; SpO_2_, saturation of peripheral oxygen.

### Within-group analysis

#### Saturation of peripheral oxygen

**Figure 2:**
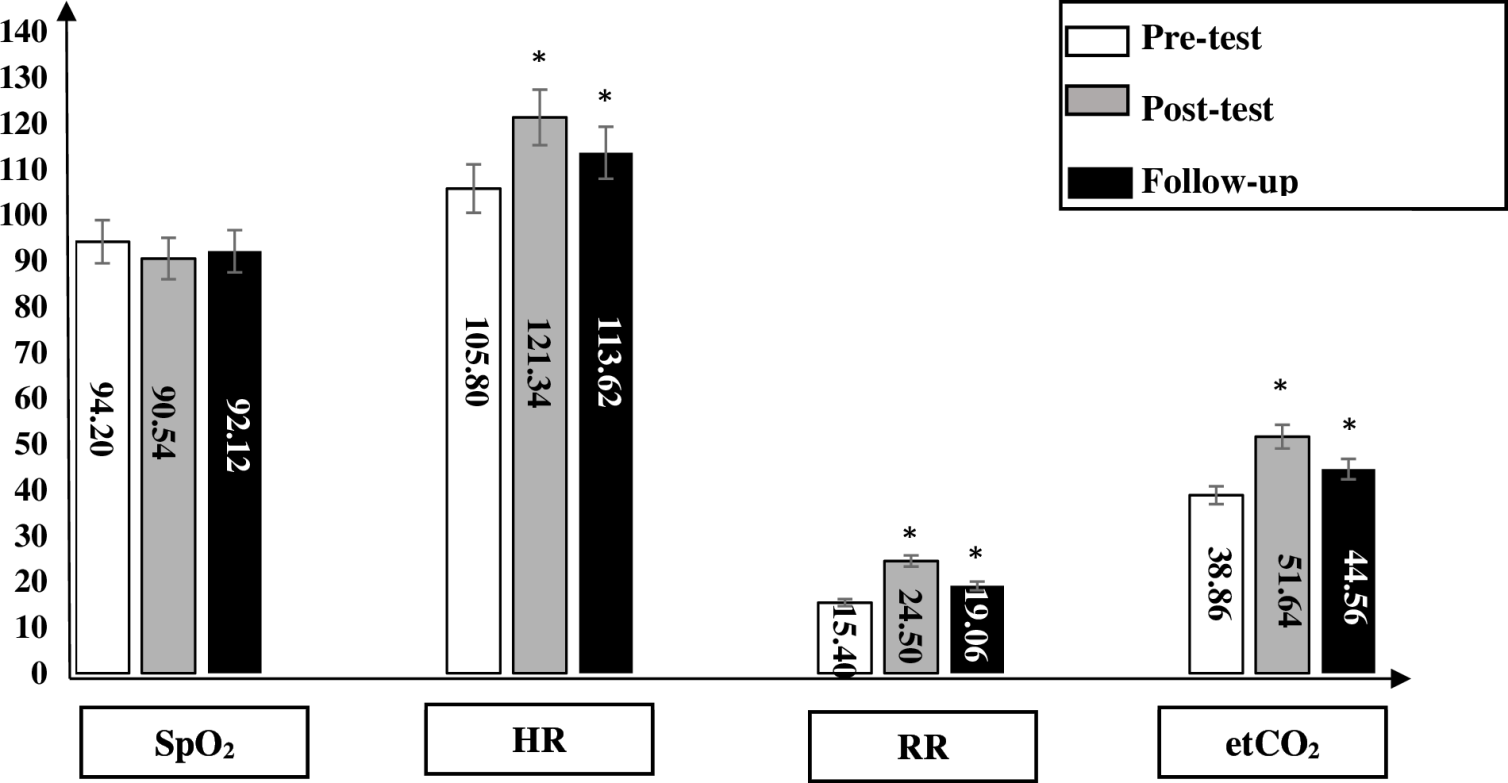
In group 1, the saturation of peripheral oxygen (SpO_2_) significantly decreased immediately after physical activity in comparison to the baseline measurements (p>0.05). After 15 min, the SpO_2_ increased; however, it stayed significant in comparison to the baseline measurements. The heart rate (HR) significantly increased immediately after physical activity in comparison to the baseline measurements (p>0.05). After 15 min, the HR decreased; however, it stayed significant in comparison to the baseline measurements. The respiratory rate (RR) significantly increased immediately after physical activity in comparison to the baseline measurements (p>0.05). After 15 min, the RR decreased; however, it stayed significant in comparison to the baseline measurements. The end-tidal carbon dioxide (etCO_2_) significantly increased immediately after physical activity in comparison to the baseline measurements (p>0.05). After 15 min, the etCO_2_ decreased; however, it stayed significant in comparison to the baseline measurements. *significant (p<0.05).

In all groups, the SpO_2_ significantly decreased immediately after physical activity in comparison to the baseline measurements (p>0.05). After 15 min, the SpO_2_ increased; however, it stayed significant in comparison to the baseline measurements. In group 1, the mean differences between the baseline and after physical activity and follow-up were 4.20% and 2.62%, respectively. In group 2, the mean differences between the baseline and after physical activity and follow-up were 3.96% and 2.50%, respectively. In group 3, the mean differences between the baseline and after physical activity and follow-up were 11.10% and 3.68%, respectively. In group 4, the mean differences between the baseline and after physical activity and follow-up were 3.62% and 2.20%, respectively. The mean difference, SD, significance and CI at 95% for the SpO_2_ within all groups are shown in table 4 and figure 2.

**Table 4:** Within-group analysis for group 2 for SpO_2_, HR, RR and etCO_2_

Measure	(I) Factor 1	(J) Factor 1	MD (I–J)	SE	P value	95% CI
SpO_2_ (%)	Pretest	Post-test	3.96	0.43	<0.001*	2.93:5.00
		Follow-up	2.50	0.43	<0.001*	1.46:3.54
HR (ppm)	Pretest	Post-test	10.26	1.86	<0.001*	14.76:5.76
		Follow-up	14.76	5.76	<0.001*	5.76:14.76
RR (bpm)	Pretest	Post-test	7.77	0.41	<0.001*	6.78:8.74
		Follow-up	1.80	0.33	<0.001*	1.01:2.60
etCO_2_ (%)	Pretest	Post-test	12.58	0.76	<0.001*	10.74:14.42
		Follow-up	6.10	0.80	0.003*	0.55:1.86

CI denotes confidence interval for difference. P value denotes significance level.

*The mean difference is significant at the 0.05 level.

bpm, breaths per minute; etCO_2_, end-tidal carbon dioxide; HR, heart rate; MD, mean difference; ppm, pulse per minute; RR, respiratory rate; SpO_2_, saturation of peripheral oxygen.

#### Heart rate

**Figure 3:**
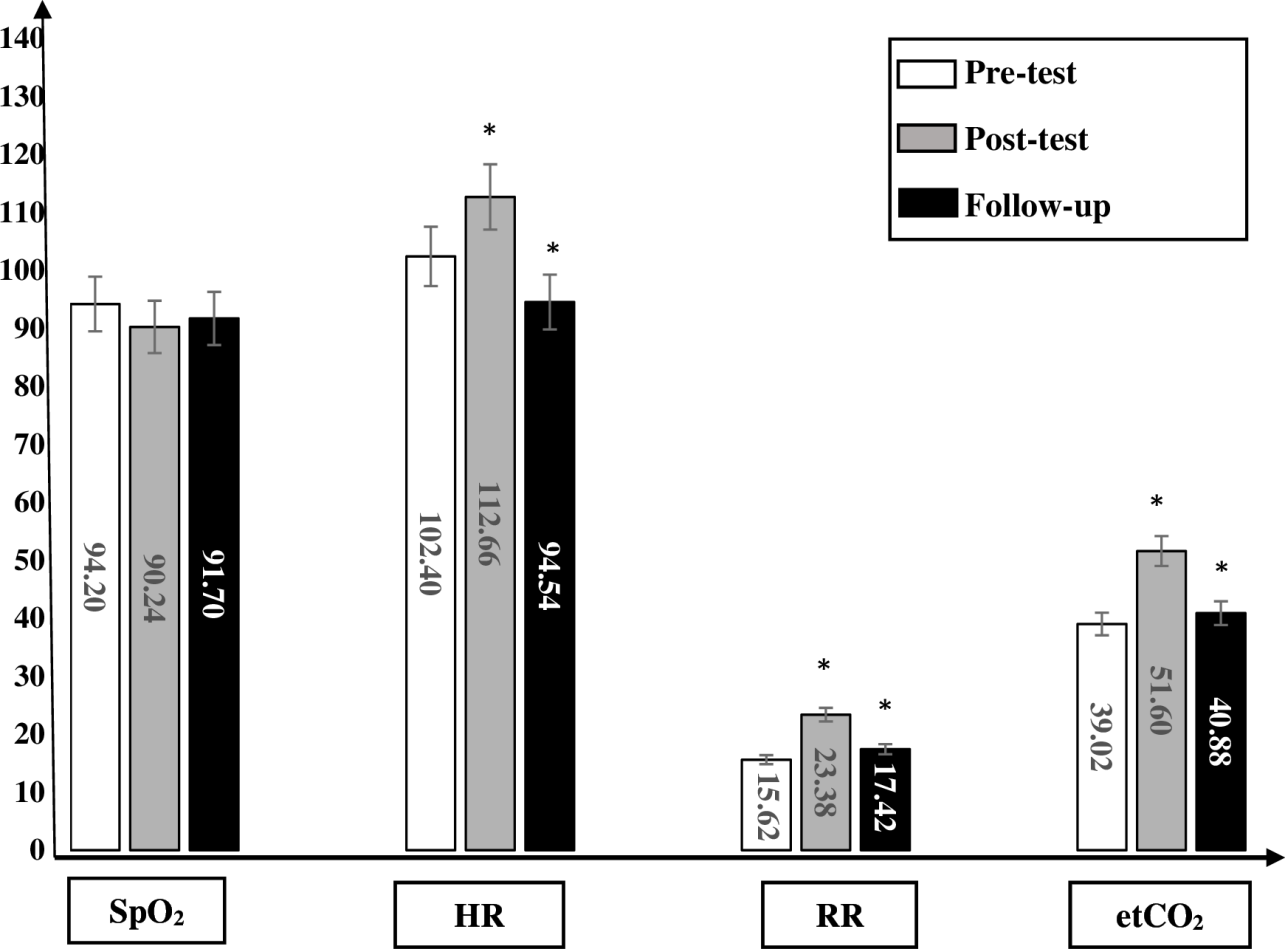
In group 2, the saturation of peripheral oxygen (SpO_2_) significantly decreased immediately after physical activity in comparison to the baseline measurements (p>0.05). After 15 min, the SpO_2_ increased; however, it stayed significant in comparison to the baseline measurements. The heart rate (HR) significantly increased immediately after physical activity in comparison to the baseline measurements (p>0.05). After 15 min, the HR decreased; however, it stayed significant in comparison to the baseline measurements. The respiratory rate (RR) significantly increased immediately after physical activity in comparison to the baseline measurements (p>0.05). After 15 min, the RR decreased; however, it stayed significant in comparison to the baseline measurements. The end-tidal carbon dioxide (etCO_2_) significantly increased immediately after physical activity in comparison to the baseline measurements (p>0.05). After 15 min, the etCO_2_ decreased; however, it stayed significant in comparison to the baseline measurements. *significant (p<0.05).

In all groups, the HR significantly increased immediately after physical activity in comparison to the baseline measurements (p>0.05). After 15 min, the HR decreased; however, it stayed significant in comparison to the baseline measurements. In group 1, the mean differences between the baseline and after physical activity and follow-up were 15.54 ppm and 7.82 ppm, respectively. In group 2, the mean differences between the baseline and after physical activity and follow-up were 10.26 ppm and 14.76 ppm, respectively. In group 3, the mean differences between the baseline and after physical activity and follow-up were 8.60 ppm and 9.38 ppm, respectively. In group 4, the mean differences between the baseline and after physical activity and follow-up were 12.20 ppm and 4.70 ppm, respectively. The mean difference, SD, significance and CI at 95% for the HR within all groups are shown in table 5 and figure 3.

**Table 5:** Within-group analysis for group 1 for SpO_2_, HR, RR and etCO_2_

Measure	(I) Factor 1	(J) Factor 1	MD (I–J)	SE	P value	95% CI
SpO_2_ (%)	Pretest	Post-test	4.20	0.43	<0.001*	3.17:5.23
		Follow-up	2.62	0.43	<0.001*	1.60:3.66
HR (ppm)	Pretest	Post-test	15.54	1.86	<0.001*	11.04:20.04
		Follow-up	7.82	1.41	<0.001*	4.42:11.21
RR (bpm)	Pretest	Post-test	9.10	0.41	<0.001*	8.12:10.08
		Follow-up	3.66	0.33	<0.001*	2.87:4.45
etCO_2_ (%)	Pretest	Post-test	12.78	0.76	<0.001*	10.94:14.62
		Follow-up	5.70	0.55	<0.001*	4.37:7.04

CI denotes confidence interval for difference. P value denotes significance level.

*The mean difference is significant at the 0.05 level.

bpm, breaths per minute; etCO_2_, end-tidal carbon dioxide; HR, heart rate; MD, mean difference; ppm, pulse per minute; RR, respiratory rate; SpO_2_, saturation of peripheral oxygen.

#### Respiratory rate

**Figure 4:**
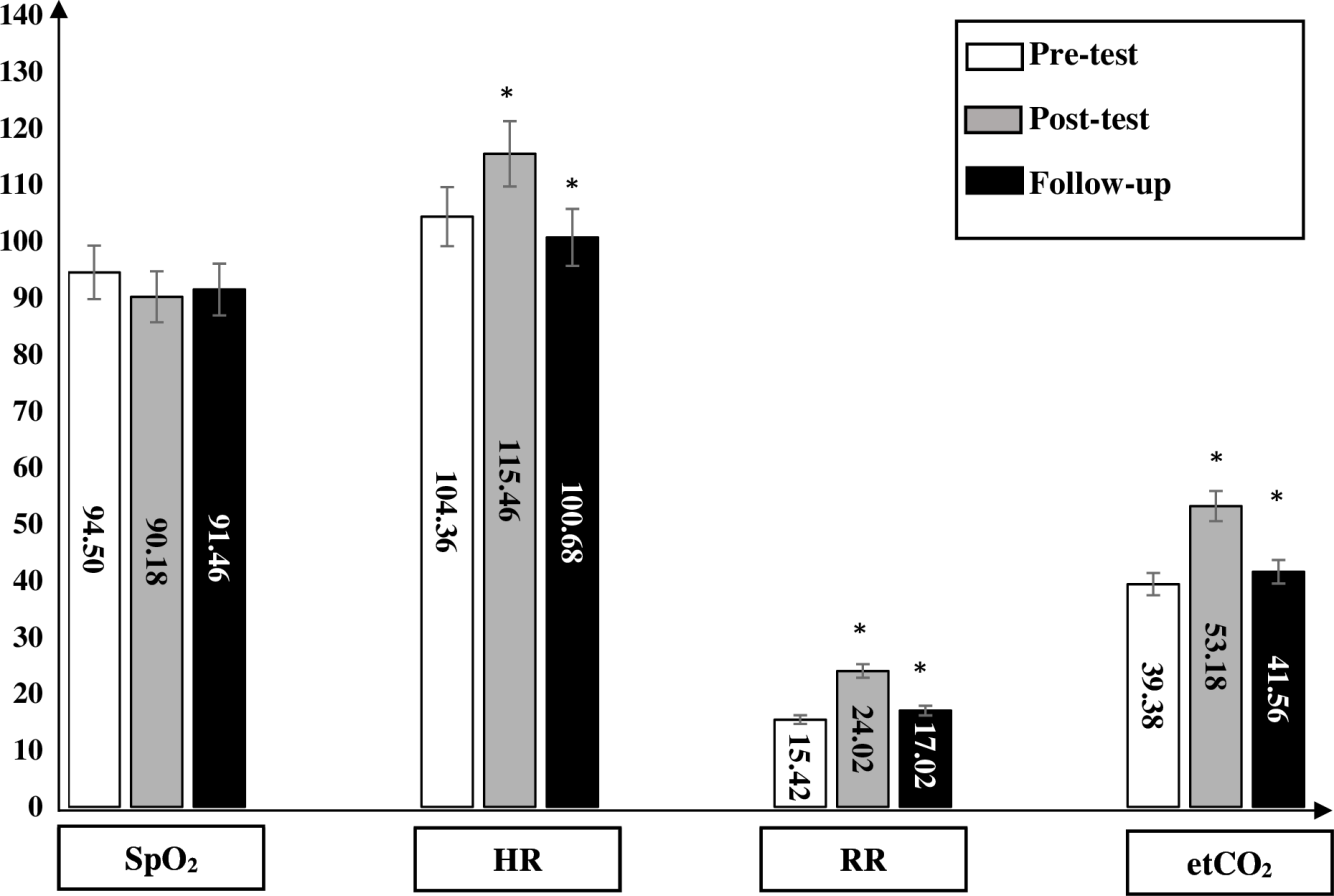
In group 3, the saturation of peripheral oxygen (SpO_2_) significantly decreased immediately after physical activity in comparison to the baseline measurements (p>0.05). After 15 min, the SpO_2_ increased; however, it stayed significant in comparison to the baseline measurements. The heart rate (HR) significantly increased immediately after physical activity in comparison to the baseline measurements (p>0.05). After 15 min, the HR decreased; however, it stayed significant in comparison to the baseline measurements. The respiratory rate (RR) significantly increased immediately after physical activity in comparison to the baseline measurements (p>0.05). After 15 min, the RR decreased; however, it stayed significant in comparison to the baseline measurements. The end-tidal carbon dioxide (etCO_2_) significantly increased immediately after physical activity in comparison to the baseline measurements (p>0.05). After 15 min, the etCO_2_ decreased; however, it stayed significant in comparison to the baseline measurements. *significant (p<0.05).

In all groups, the RR significantly increased immediately after physical activity in comparison to the baseline measurements (p>0.05). After 15 min, the RR decreased; however, it stayed significant in comparison to the baseline measurements. In group 1, the mean differences between the baseline and after physical activity and follow-up were 9.10 bpm and 3.66 bpm, respectively. In group 2, the mean differences between the baseline and after physical activity and follow-up were 7.77 bpm and 1.80 bpm, respectively. In group 3, the mean differences between the baseline and after physical activity and follow-up were 8.60 bpm and 1.60 bpm, respectively. In group 4, the mean differences between the baseline and after physical activity and follow-up were 8.88 bpm and 0.940 bpm, respectively. The mean difference, SD, significance and CI at 95% for the RR within all groups are shown in table 6 and figure 4.

**Table 6:** Within-group analysis for group 3 for SpO_2_, HR, RR and etCO_2_

Measure	(I) Factor 1	(J) Factor 1	MD (I–J)	SE	P value	95% CI
SpO_2_ (%)	Pretest	Post-test	11.10	1.86	<0.001*	6.60:15.60
		Follow-up	3.68	1.41	0.029*	0.29:7.07
HR (ppm)	Pretest	Post-test	8.60	0.41	<0.001*	19.43:16.61
		Follow-up	9.38	0.52	<0.001*	10.43:8.33
RR (bpm)	Pretest	Post-test	8.60	0.34	<0.001*	7.62:9.58
		Follow-up	1.60	0.33	<0.001*	0.81:2.39
etCO_2_ (%)	Pretest	Post-test	13.80	0.76	<0.001*	11.96:15.64
		Follow-up	2.18	0.55	<0.001*	0.85:3.52

CI denotes confidence interval for difference. P value denotes significance level.

*The mean difference is significant at the 0.05 level.

bpm, breaths per minute; etCO_2_, end-tidal carbon dioxide; HR, heart rate; MD, mean difference; ppm, pulse per minute; RR, respiratory rate; SpO_2_, saturation of peripheral oxygen.

#### End-tidal carbon dioxide

**Figure 5:**
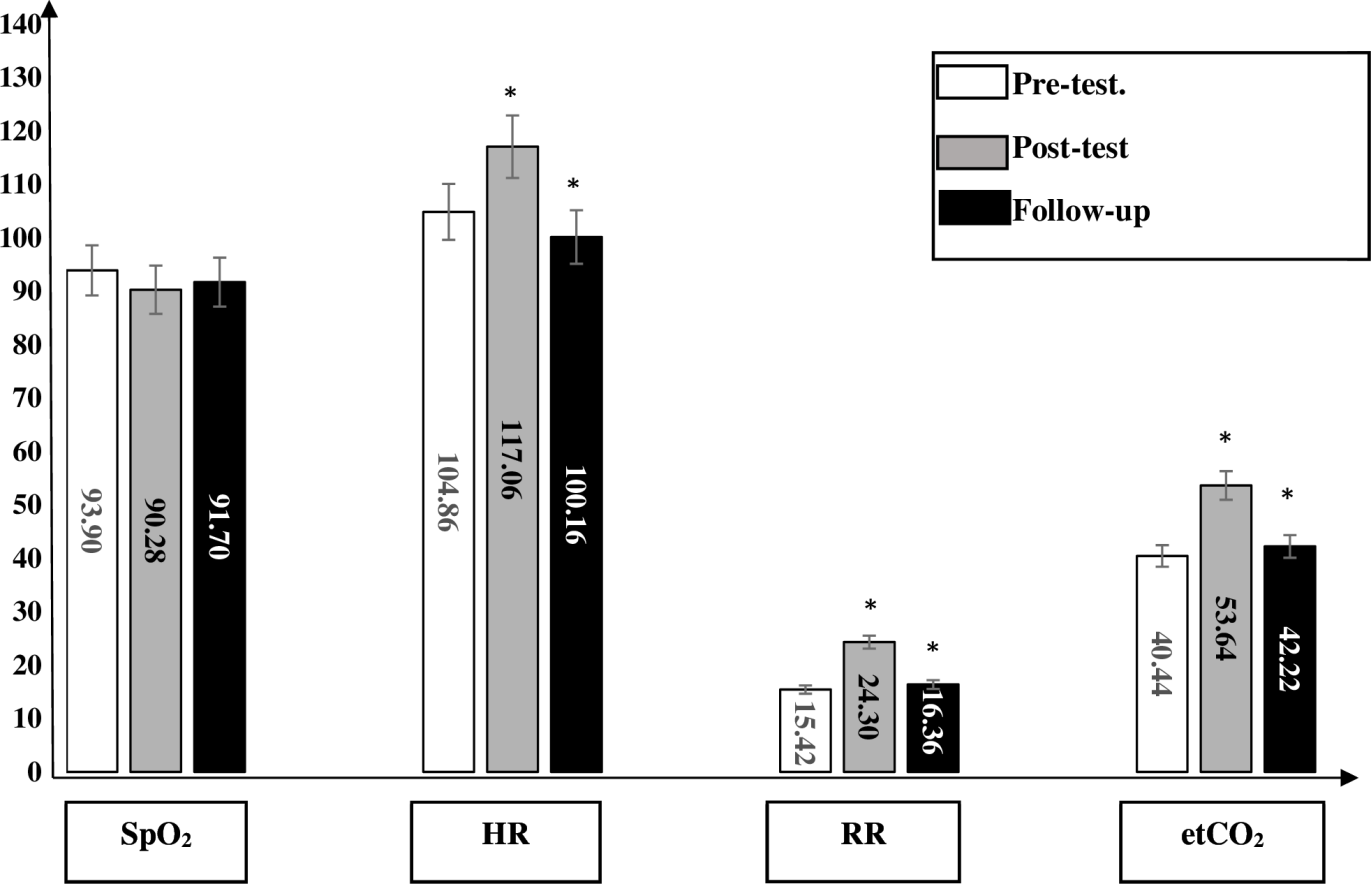
In group 4, the saturation of peripheral oxygen (SpO_2_) significantly decreased immediately after physical activity in comparison to the baseline measurements (p>0.05). After 15 min, the SpO_2_ increased; however, it stayed significant in comparison to the baseline measurements. The heart rate (HR) significantly increased immediately after physical activity in comparison to the baseline measurements (p>0.05). After 15 min, the HR decreased; however, it stayed significant in comparison to the baseline measurements. The respiratory rate (RR) significantly increased immediately after physical activity in comparison to the baseline measurements (p>0.05). After 15 min, the RR decreased; however, it stayed significant in comparison to the baseline measurements. The end-tidal carbon dioxide (etCO_2_) significantly increased immediately after physical activity in comparison to the baseline measurements (p>0.05). After 15 min, the etCO_2_ decreased; however, it stayed significant in comparison to the baseline measurements. *significant (p<0.05).

In all groups, the etCO_2_ significantly increased immediately after physical activity in comparison to the baseline measurements (p>0.05). After 15 min, the etCO_2_ decreased; however, it stayed significant in comparison to the baseline measurements. In group 1, the mean differences between the baseline and after physical activity and follow-up were 12.78% and 5.70%, respectively. In group 2, the mean differences between the baseline and after physical activity and follow-up were 12.58% and 6.10%, respectively. In group 3, the mean differences between the baseline and after physical activity and follow-up were 13.80% and 2.18%, respectively. In group 4, the mean differences between the baseline and after physical activity and follow-up were 13.20% and 1.780%, respectively. The mean difference, SD, significance and CI at 95% for the etCO_2_ within all groups are shown in table 7 and figure 5.

**Table 7:** Within-group analysis for group 4 for SpO_2_, HR, RR and etCO_2_

Measure	(I) Factor 1	(J) Factor 1	MD (I–J)	SE	P value	95% CI
SpO_2_ (%)	Pretest	Post-test	3.62	0.43	<0.001*	2.59:4.65
		Follow-up	2.20	0.43	<0.001*	1.16:3.24
HR (ppm)	Pretest	Post-test	12.20	1.86	<0.001*	7.70:16.70
		Follow-up	4.70	1.41	0.003*	1.31:8.09
RR (bpm)	Pretest	Post-test	8.88	0.41	<0.001*	7.90:9.86
		Follow-up	0.940	0.33	0.014*	0.150:1.73
etCO_2_ (%)	Pretest	Post-test	13.20	0.76	<0.001*	11.36:15.04
		Follow-up	1.780	0.55	0.005*	0.45:3.12

CI denotes confidence interval for difference. P value denotes significance level.

*The mean difference is significant at the 0.05 level.

bpm, breaths per minute; etCO_2_, end-tidal carbon dioxide; HR, heart rate; MD, mean difference; ppm, pulse per minute; RR, respiratory rate; SpO_2_, saturation of peripheral oxygen.

## Discussion

This study was conducted to compare the immediate effect of physical activity on the ANS in individuals with ASD of different age groups. This study measured SpO_2_, HR, RR and etCO_2_ parameters in different age groups among individuals with ASD. This study is unique as it was the first study that investigated and compared the ANS response among different age groups of individuals with ASD. This study was the first study to investigate the effect of aerobic physical activity on SpO_2_ and etCO_2_ in individuals with ASD. Also, this study measured the lasting effect of physical activity on the ANS. We found that the SpO_2_ significantly decreased immediately after the physical activity, while HR, RR and etCO_2_ significantly increased immediately after physical activity in comparison to the baseline measurements. Contrary to other ANS parameters (SpO_2_, RR and etCO_2_), HR in early ages (4–7 years old) was higher after physical activity and remained elevated longer than other ages.

### Between-group comparisons

Immediately after physical activity, there were non-significant differences between all groups for all outcome measurements (p>0.05) except for the HR; while at follow-up (after 15 min of rest), there were significant differences between group 1 and other groups for all outcome measurements (p<0.05), and there were non-significant differences between the other three groups (p>0.05). Immediately after physical activity, the highest mean difference in SpO_2_, HR, RR and etCO_2_ was between group 1 and group 3 (0.36%), group 1 and group 2 (8.68* ppm), group 1 and group 2 (1.12 bpm), and group 2 and group 3 (2.04%), respectively. At follow-up (after 15 min of rest), the highest mean difference in SpO_2_, HR, RR and etCO_2_ was between group 1 and group 3 (0.66*%), group 1 and group 2 (19.08* ppm), group 1 and group 4 (2.70 bpm), and group 1 and group 2 (3.68*%), respectively.

These results might be attributed to the majority of children with ASD who have congenital heart diseases (CHD) in the form of atrial or ventricular septal defect.^39^ This atrial or ventricular septal defect decreases cardiac output which causes the heart to work less efficiently. Therefore, the HR response to physical activity may be affected to compensate for the weak response of the heart. Consequently, the usual recovery after stopping physical activity would be affected to return the heart to a normal state in the shortest possible time.^39^ The abnormal response in HR leads to abnormal responses in SpO_2_, RR and etCO_2_.

This is matched with the study by Fioriello *et al*.^40^ They found that HR was more elevated and less modulated in children with ASD, especially with young children with a mean age of 3–4 years. At follow-up (after 15 min of rest), the mean in group 1 was significantly higher than other groups for all outcome measurements (HR, SpO_2_, RR, etCO_2_), while there were non-significant differences between the other three groups. This finding is close to the study by Sigmon *et al* who investigated the association between CHD and ASD in cases with an average age of 7 years.^41^ They found that a high percentage of children with CHD had ASD with an average age of 7. Another recent meta-analysis reached close findings as it investigated the link between CHD and ASD in children.^42^ They screened 250 611 subjects (3984 CHD, 9829 ASD and 236 798 controls). They found that CHD increases the risk of developing ASD.

### Saturation of peripheral oxygen

There was a significant decrease in SpO_2_ in all groups after physical activity, and this decrease continued to be significant at follow-up (after 15 min of rest) in comparison to the baseline data. This decrease might be attributed to the increase in the demand of the working muscles for oxygen during physical activity.^43^ During physical activity, the working muscles need more oxygen to continue working and avoid the accumulation of lactic acid and the development of fatigue.^23 44^ Children with ASD have less SpO_2_ than TD children, thus their ability to continue physical activity for a long time will be less in comparison to TD children.^25^

Ando *et al* came to the same results of this study.^45^ They investigated the effect of moderate-intensity physical activity (to 60% of peak oxygen uptake) on SpO_2_. They found that moderate-intensity physical activity significantly decreased SpO_2_.^45^ Also, Eroğlu *et al* investigated the acute effect of aerobic physical activity on SpO_2_. They applied aerobic physical activity for 90 min for 6 days/week. They found that aerobic physical activity significantly reduced SpO_2_ in arterial blood.^46^ A similar finding was reached by Coli *et al* who investigated the effect of moderate-intensity physical activity on SpO_2_ in obese females.^47^ They used 40 min of walking using a treadmill. They found that aerobic physical activity significantly reduced the SpO_2_.

### Heart rate

There was a significant increase in HR in all groups after physical activity, and this increase continued to be significant at follow-up (after 15 min of rest) in comparison to the baseline data. The increase in HR might be attributed to the increased demand for blood by the working muscles,^48^ because the ANS has a major responsibility in controlling the cardiovascular reactions to physical activity needed by the raised metabolic demand of the working skeletal muscles. Physical activity is associated with a well-known decrease in cardiac parasympathetic action and a rise in cardiac sympathetic action. This leads to an increase in HR, cardiac output and stroke volume to redistribute the bloodstream to the working skeletal muscles.^48 49^ After 15 min of rest, the increase in the HR remained significant in comparison to baseline data which might be attributed to the children with ASD who have reduced cardiorespiratory capacity and lesser HR response in comparison to controls during physical activity.^18 22^

The results of Schiffer *et al*^50^ and Greiwe and Kohrt^51^ were similar to the current study. They investigated the effect of Nordic walking, walking and jogging on HR in normal subjects.^50^ They found that Nordic walking, walking and jogging significantly increased HR and it was higher in Nordic walking and walking than jogging. Flynn *et al* investigated the response of the heart to forward walking and running to backward walking and running^52^ in normal subjects. They found that HR increased in both physical activity types, and this increase in HR was more during backward walking and running than during forward walking and running. Pace and Bricout conducted a study to compare the effect of physical activity on HR between children with ASD and TD children.^18^ They found that both groups showed a significant increase in HR with a lower increase in children with ASD.

### Respiratory rate

There was a significant increase in RR in all groups after physical activity, and this increase continued to be significant at follow-up (after 15 min of rest) in comparison to baseline data. In physical activity, muscles work harder. Thus, the body uses more oxygen and produces more carbon dioxide. The RR, alveolar ventilation and alveolar-capillary diffusion rise with the rise in metabolic rate to inhibit the increase in CO_2_ and decrease in O_2_. Breathing must increase from around 15 times a minute (12 L of air) during resting to around 40–60 times a minute (100 L of air) during physical activity. Also, the increase in RR and alveolar ventilation is necessary to remove a part of the raised heat created by physical activity. This can be accomplished by rising ventilation of the conducting airways (dead space ventilation).^53 54^

The results of Berry *et al* reached the same results as this current study.^55^ They compared the increase in ventilation in runners during running with walking at parallel metabolic rates. They found that ventilation parameters were significantly increased in both groups. The minute ventilation was significantly more throughout running as compared with walking, while alveolar ventilation was not significantly different between running and walking. Also, dead space ventilation was significantly more throughout running as compared with walking. McMurray and Ahlborn compared respiratory responses between running and walking at similar metabolic rates.^56^ They found that respiratory response increased in both groups with more increase in running than walking. Furthermore, Bricout *et al* investigated the cardiorespiratory capacity in children with ASD in comparison to TD children.^22^ They found that aerobic capacity values (VO_2_ peak), effort time and maximum velocity increased in both groups; however, they were significantly lesser in comparison to TD children.

### End-tidal carbon dioxide

There was a significant decrease in etCO_2_ in all groups after physical activity, and this decrease continued to be significant at follow-up (after 15 min of rest) in comparison to the baseline data. The etCO_2_ is the level of CO_2_ that is produced at the terminal of an exhaled air. The etCO_2_ level indicates the competence with which CO_2_ is transferred in the circulation back to the lungs.^57^ During physical activity, the etCO_2_ decreases. The etCO_2_ decreases in physical activity due to high ventilation/perfusion ratio mismatching and it indicates both diminished cardiac output reaction to physical activity and increased respiratory ventilation.^58^

The results of Matsumoto *et al* reached the same results of this study. They investigated the response of etCO_2_ to physical activity in patients with heart failure.^59^ They found that etCO_2_ significantly decreased during physical activity. A similar result was reached by Tanabe et al who investigated the importance of etCO2 reaction to physical activity and its relative to functional capacity in patients with chronic heart failure.^58^ They found that physical activity significantly decreased etCO_2_ and it had a strong relation to functional capacity. Also, Myers *et al* investigated the effect of etCO_2_ and cardiac performance during physical activity in patients with heart failure.^60^ They found that etCO_2_ significantly lowered during physical activity and it reflected the severity of heart failure.

### Limitations

This study was limited to several factors. We just performed a 1-hour walk one time to test the immediate effect of physical activity on the ANS in children with ASD. Also, this study did not include a control group because comparing the effect of physical activity on the ANS in children with ASD to normally developed children was performed in earlier studies. Future studies are recommended to test the effect of prolonged performance of physical activity on the ANS in children with ASD of different ages. This study used a specific physical activity programme for all groups; however, the responses of participants might be different because this study aimed to test the effect of intense physical activity on their aerobic capacity.

## Conclusion

This study revealed that the SpO_2_ significantly decreased immediately after the physical activity, while HR, RR and etCO_2_ significantly increased immediately after physical activity in comparison to the baseline measurements. Contrary to other ANS parameters (SpO_2_, RR and etCO_2_), HR in early ages (4–7 years old) was higher after physical activity and remained elevated longer than other ages. The early ages (4–7 years old) take more time to return to the normal status of ANS parameters including SpO_2_, HR, RR and etCO_2_.

## Data Availability

Data are available upon reasonable request by emailing the corresponding author.
