# Supplementary material for: Immediate effect of physical activity on the autonomic nervous system in individuals with autism spectrum disorders of different age groups: a randomised trial
Source: BMJ Open Sport Exerc Med. 2024 Apr 11;10(2):e001822. doi: 10.1136/bmjsem-2023-001822 (PMC11015250; doi:10.1136/bmjsem-2023-001822)
Supplement: Supplementary data [file bmjsem-2023-001822supp001.pdf]

Supplementary Table 1: Between-groups analysis for HRV, HR, RR, and ECO_2

Measure	Time	(I) Age Groups	(J) Age Groups	MD (I-J)	SE	P	95% CI Lower Bound	95% CI Upper Bound
SPO2	Pre-test	G1_4_7yrs	G2_7_10 yrs	.54	.57	1.00	-.97-	2.05
			G3_10_13yrs	.24	.57	1.00	-1.27-	1.75
			G4_13_18yrs	.84	.57	.84	-.67-	2.35
		G2_7_10 yrs	G1_4_7yrs	-.54-	.57	1.00	-2.05-	.97
			G3_10_13yrs	-.30-	.57	1.00	-1.81-	1.21
			G4_13_18yrs	.30	.57	1.00	-1.21-	1.81
		G3_10_13yrs	G1_4_7yrs	-.24-	.57	1.00	-1.75-	1.27
			G2_7_10 yrs	.30	.57	1.00	-1.21-	1.81
			G4_13_18yrs	.60	.57	1.00	-.91-	2.11
		G4_13_18yrs	G1_4_7yrs	-.84-	.57	.84	-2.35-	.67
			G2_7_10 yrs	-.30-	.57	1.00	-1.81-	1.21
			G3_10_13yrs	-.60-	.57	1.00	-2.11-	.91
	Post-test	G1_4_7yrs	G2_7_10 yrs	.30	.16	.40	-.13-	.73
			G3_10_13yrs	.36	.16	.17	-.07-	.79
			G4_13_18yrs	.26	.16	.66	-.17-	.69
		G2_7_10 yrs	G1_4_7yrs	-.30-	.16	.40	-.73-	.13
			G3_10_13yrs	.06	.16	1.00	-.37-	.49
			G4_13_18yrs	-.04-	.16	1.00	-.47-	.39
		G3_10_13yrs	G1_4_7yrs	-.36-	.16	.17	-.79-	.07
			G2_7_10 yrs	-.06-	.16	1.00	-.49-	.37
			G4_13_18yrs	-.10-	.16	1.00	-.53-	.33
		G4_13_18yrs	G1_4_7yrs	-.26-	.16	.66	-.69-	.17
			G2_7_10 yrs	.04	.16	1.00	-.39-	.47

Ahmed MM, *et al. BMJ Open Sp Ex Med* 2024; 10:e001822. doi: 10.1136/bmjsem-2023-001822

Supplemental material BMJ Publishing Group Limited (BMJ) disclaims all liability and responsibility arising from any reliance placed on this supplemental material which has been supplied by the author(s) BMJ Open Sp Ex Med

			G3_10_13yrs	.10	.16	1.00	-.33-	.53
	Follow-up	G1_4_7yrs	G2_7_10 yrs	.42*	.15	.04	.02	.82
			G3_10_13yrs	.66*	.15	.00	.26	1.06
			G4_13_18yrs	.42*	.15	.04	.02	.82
		G2_7_10 yrs	G1_4_7yrs	-.42-*	.15	.04	-.82-	-.02-
			G3_10_13yrs	.12	.15	.69	-.16-	.64
			G4_13_18yrs	.05	.15	1.00	-.40-	.40
		G3_10_13yrs	G1_4_7yrs	-.66-*	.15	.00	-1.06-	-.26-
			G2_7_10 yrs	-.24-	.15	.69	-.64-	.16
			G4_13_18yrs	-.24-	.15	.69	-.64-	.16
		G4_13_18yrs	G1_4_7yrs	-.42-*	.15	.04	-.82-	-.02-
			G2_7_10 yrs	-.05	.15	1.00	-.40-	.40
			G3_10_13yrs	.12	.15	.69	-.16-	.64
HR	Pre-test	G1_4_7yrs	G2_7_10 yrs	3.40	1.73	.30	-1.21-	8.01
			G3_10_13yrs	1.44	1.73	1.00	-3.17-	6.05
			G4_13_18yrs	.94	1.73	1.00	-3.67-	5.55
		G2_7_10 yrs	G1_4_7yrs	-3.40-	1.73	.30	-8.01-	1.21
			G3_10_13yrs	-1.96-	1.73	1.00	-6.57-	2.65
			G4_13_18yrs	-2.46-	1.73	.94	-7.07-	2.15
		G3_10_13yrs	G1_4_7yrs	-1.44-	1.73	1.00	-6.05-	3.17
			G2_7_10 yrs	1.96	1.73	1.00	-2.65-	6.57
			G4_13_18yrs	-.50-	1.73	1.00	-5.11-	4.11
		G4_13_18yrs	G1_4_7yrs	-.94-	1.73	1.00	-5.55-	3.67
			G2_7_10 yrs	2.46	1.73	.94	-2.15-	7.07
			G3_10_13yrs	.50	1.73	1.00	-4.11-	5.11
	Post-test	G1_4_7yrs	G2_7_10 yrs	8.68*	2.61	.01	1.73	15.63
			G3_10_13yrs	5.88	2.61	.15	-1.07-	12.83

Ahmed MM, *et al. BMJ Open Sp Ex Med* 2024; 10:e001822. doi: 10.1136/bmjsem-2023-001822

			G4_13_18yrs	4.28	2.61	.61	-2.67-	11.23
		G2_7_10 yrs	G1_4_7yrs	-8.68-*	2.61	.01	-15.63-	-1.73-
			G3_10_13yrs	-2.80-	2.61	1.00	-9.75-	4.15
			G4_13_18yrs	-4.40-	2.61	.56	-11.35-	2.55
		G3_10_13yrs	G1_4_7yrs	-5.88-	2.61	.15	-12.83-	1.07
			G2_7_10 yrs	2.80	2.61	1.00	-4.15-	9.75
			G4_13_18yrs	-1.60-	2.61	1.00	-8.55-	5.35
		G4_13_18yrs	G1_4_7yrs	-4.28-	2.61	.61	-11.23-	2.67
			G2_7_10 yrs	4.40	2.61	.56	-2.55-	11.35
			G3_10_13yrs	1.60	2.61	1.00	-5.35-	8.55
	**Follow-up**	G1_4_7yrs	G2_7_10 yrs	19.08*	2.20	.00	13.22	24.94
			G3_10_13yrs	12.94*	2.20	.00	7.08	18.80
			G4_13_18yrs	13.46*	2.20	.00	7.60	19.32
		G2_7_10 yrs	G1_4_7yrs	-19.08-*	2.20	.00	-24.94-	-13.22-
			G3_10_13yrs	-6.14-*	2.20	.03	-12.00-	-.28-
			G4_13_18yrs	-5.62-	2.20	.07	-11.48-	.24
		G3_10_13yrs	G1_4_7yrs	-12.94-*	2.20	.00	-18.80-	-7.08-
			G2_7_10 yrs	6.14*	2.20	.03	.28	12.00
			G4_13_18yrs	.52	2.20	1.00	-5.34-	6.38
		G4_13_18yrs	G1_4_7yrs	-13.46-*	2.20	.00	-19.32-	-7.60-
			G2_7_10 yrs	5.62	2.20	.07	-.24-	11.48
			G3_10_13yrs	-.52-	2.20	1.00	-6.38-	5.34
RR	**Pre-test**	G1_4_7yrs	G2_7_10 yrs	-.22-	.24	1.00	-.86-	.42
			G3_10_13yrs	-.02-	.24	1.00	-.66-	.62
			G4_13_18yrs	-.02-	.24	1.00	-.66-	.62
		G2_7_10 yrs	G1_4_7yrs	.22	.24	1.00	-.42-	.86
			G3_10_13yrs	.20	.24	1.00	-.44-	.84

Ahmed MM, *et al. BMJ Open Sp Ex Med* 2024; 10:e001822. doi: 10.1136/bmjsem-2023-001822

BMJ Publishing Group Limited (BMJ) disclaims all liability and responsibility arising from any reliance placed on this supplemental material which has been supplied by the author(s)

		G4_13_18yrs	.20	.24	1.00	-.44-	.84
	G3_10_13yrs	G1_4_7yrs	.02	.24	1.00	-.62-	.66
		G2_7_10 yrs	-.20-	.24	1.00	-.84-	.44
		G4_13_18yrs	-1.78	.24	1.00	-.64-	.64
	G4_13_18yrs	G1_4_7yrs	.02	.24	1.00	-.62-	.66
		G2_7_10 yrs	-.20-	.24	1.00	-.84-	.44
		G3_10_13yrs	1.78E-17	.24	1.00	-.64-	.64
**Post-test**	G1_4_7yrs	G2_7_10 yrs	1.12	.58	.34	-.43-	2.67
		G3_10_13yrs	.48	.58	1.00	-1.07-	2.03
		G4_13_18yrs	.20	.58	1.00	-1.35-	1.75
	G2_7_10 yrs	G1_4_7yrs	-1.12-	.58	.34	-2.67-	.43
		G3_10_13yrs	-.64-	.58	1.00	-2.19-	.91
		G4_13_18yrs	-.92-	.58	.70	-2.47-	.63
	G3_10_13yrs	G1_4_7yrs	-.48-	.58	1.00	-2.03-	1.07
		G2_7_10 yrs	.64	.58	1.00	-.91-	2.19
		G4_13_18yrs	-.28-	.58	1.00	-1.83-	1.27
	G4_13_18yrs	G1_4_7yrs	-.20-	.58	1.00	-1.75-	1.35
		G2_7_10 yrs	.92	.58	.70	-.63-	2.47
		G3_10_13yrs	.28	.58	1.00	-1.27-	1.83
**Follow-up**	G1_4_7yrs	G2_7_10 yrs	1.64*	.46	.00	.41	2.87
		G3_10_13yrs	2.04*	.46	.00	.81	3.27
		G4_13_18yrs	2.70*	.46	.00	1.47	3.93
	G2_7_10 yrs	G1_4_7yrs	-1.64-*	.46	.00	-2.87-	-.41-
		G3_10_13yrs	.40	.46	1.00	-.83-	1.63
		G4_13_18yrs	1.06	.46	.14	-.17-	2.29
	G3_10_13yrs	G1_4_7yrs	-2.04-*	.46	.00	-3.27-	-.81-
		G2_7_10 yrs	-.40-	.46	1.00	-1.63-	.83

Ahmed MM, *et al. BMJ Open Sp Ex Med* 2024; 10:e001822. doi: 10.1136/bmjsem-2023-001822

			G4_13_18yrs	.66	.46	.92	-.57-	1.89
		G4_13_18yrs	G1_4_7yrs	-2.70-*	.46	.00	-3.93-	-1.47-
			G2_7_10 yrs	-1.06-	.46	.14	-2.29-	.17
			G3_10_13yrs	-.66-	.46	.92	-1.89-	.57
etCO2	Pre-test	G1_4_7yrs	G2_7_10 yrs	-.16-	.90	1.00	-2.57-	2.25
			G3_10_13yrs	-.52-	.90	1.00	-2.93-	1.89
			G4_13_18yrs	-1.58-	.90	.49	-3.99-	.83
		G2_7_10 yrs	G1_4_7yrs	.16	.90	1.00	-2.25-	2.57
			G3_10_13yrs	-.36-	.90	1.00	-2.77-	2.05
			G4_13_18yrs	-1.42-	.90	.71	-3.83-	.99
		G3_10_13yrs	G1_4_7yrs	.52	.90	1.00	-1.89-	2.93
			G2_7_10 yrs	.36	.90	1.00	-2.05-	2.77
			G4_13_18yrs	-1.06-	.90	1.00	-3.47-	1.35
		G4_13_18yrs	G1_4_7yrs	1.58	.90	.49	-.83-	3.99
			G2_7_10 yrs	1.42	.90	.71	-.99-	3.83
			G3_10_13yrs	1.06	.90	1.00	-1.35-	3.47
	Post-test	G1_4_7yrs	G2_7_10 yrs	.04	.93	1.00	-2.44-	2.52
			G3_10_13yrs	-1.54-	.93	.60	-4.02-	.94
			G4_13_18yrs	-2.00-	.93	.20	-4.48-	.48
		G2_7_10 yrs	G1_4_7yrs	-.04-	.93	1.00	-2.52-	2.44
			G3_10_13yrs	-1.58-	.93	.55	-4.06-	.90
			G4_13_18yrs	-2.04-	.93	.18	-4.52-	.44
		G3_10_13yrs	G1_4_7yrs	1.54	.93	.60	-.94-	4.02
			G2_7_10 yrs	1.58	.93	.55	-.90-	4.06
			G4_13_18yrs	-.46-	.93	1.00	-2.94-	2.02
		G4_13_18yrs	G1_4_7yrs	2.00	.93	.20	-.48-	4.48
			G2_7_10 yrs	2.04	.93	.18	-.44-	4.52

		G3_10_13yrs	.46	.93	1.00	-2.02-	2.94
Follow-up	G1_4_7yrs	G2_7_10 yrs	3.68*	.83	.00	1.47	5.89
		G3_10_13yrs	3.00*	.83	.00	.79	5.21
		G4_13_18yrs	2.34*	.83	.03	.13	4.55
	G2_7_10 yrs	G1_4_7yrs	-3.68-*	.83	.00	-5.89-	-1.47-
		G3_10_13yrs	-.68-	.83	1.00	-2.89-	1.53
		G4_13_18yrs	-1.34-	.83	.65	-3.55-	.87
	G3_10_13yrs	G1_4_7yrs	-3.00-*	.83	.00	-5.21-	-.79-
		G2_7_10 yrs	.68	.83	1.00	-1.53-	2.89
		G4_13_18yrs	-.66-	.83	1.00	-2.87-	1.55
	G4_13_18yrs	G1_4_7yrs	-2.34-*	.83	.03	-4.55-	-.13-
		G2_7_10 yrs	1.34	.83	.65	-.87-	3.55
		G3_10_13yrs	.66	.83	1.00	-1.55-	2.87

M: Mean Difference, SE: standard error, SpO2: Saturation of Peripheral Oxygen, HR: heart rate: RR: respiratory rate, etCO2: End-tidal carbon dioxide, P: significance level, *: The mean difference is significant at the .05 level, CI: Confidence Interval for Difference

For Peer Review Only
